# Socio-demographic study on extent of knowledge, awareness, attitude, and risks of zoonotic diseases among livestock owners in Puducherry region

**DOI:** 10.14202/vetworld.2016.1018-1024

**Published:** 2016-09-26

**Authors:** K. Rajkumar, A. Bhattacharya, S. David, S. Hari Balaji, R. Hariharan, M. Jayakumar, N. Balaji

**Affiliations:** Department of Veterinary Public Health and Epidemiology, Rajiv Gandhi Institute of Veterinary Education and Research, Puducherry - 605 009, India

**Keywords:** attitude, awareness, livestock farmers, risk, zoonotic disease

## Abstract

**Aim:**

This study was conducted to assess the extent of knowledge, awareness, attitude, and risks of zoonotic diseases among livestock owners in Puducherry region.

**Materials and Methods:**

A total of 250 livestock farmers were selected randomly from eight revenue villages. And each farmer was interviewed with a questionnaire containing both open- and close-ended questions on various aspects of zoonotic diseases, a total of 49 questionnaires were framed to assess the source and transmission of infection to the farmers and to test their knowledge and awareness about zoonotic diseases. The data collected were analyzed by chi-square test using software Graph pad prism, and results were used to assess the relationship between education level and zoonotic disease awareness; risk of zoonotic diseases and its relation with independent variables.

**Results:**

The present survey analysis represents that most of the respondents are belonging to the age group of 41-60 years. About 42.8% of respondents’ household having a graduate. The most of the respondent are small-scale farmers and their monthly income was less than Rs. 10,000. About 61.2% of farmers were keeping their animal shed clean. About 29.6% of the respondents were ignorant about cleaning the dog bitten wound. Only 16.4% of respondents knew that diseases in animals can be transmitted to humans. Only 4.8%, 3.6%, 6.8%, and 22.4% of respondents knew about the zoonotic potential of diseases such as brucellosis, tuberculosis (TB), anthrax, and avian flu, respectively. Only 18% of the respondents were aware about zoonotic diseases from cattle. Regarding the list of zoonotic diseases contracted, 37.7% reported respiratory infection, 31.1% digestive disturbances, 15.5% had dermatological problem, and 15.5% reported indiscrete disease such as fever, body pain, and headache joint pain. From the respondent got the zoonotic disease (n=45), 51.2% of the respondent reported chronic infection and 48.8% of the respondent reported acute form of zoonotic infection. About 30% of the respondents’ farm had an incidence of abortion. Our analyses showed that there was significant in educational level of respondents and treatment of dog bitten animals. Furthermore, there was statistical significance in occurrence of hand and foot lesions in the respondent and occurrence of foot-and-mouth disease outbreak in their animals.

**Conclusion:**

From this study, it is concluded that involvement of educated family members in farming practices can create awareness and improve knowledge toward zoonotic disease. Further creation of awareness toward zoonotic diseases is of utmost important.

## Introduction

Puducherry is a union territory located in the southern east part of India. It is a coastal region with tropical wet and dry climate. According to 19^th^ livestock census, Puducherry has a livestock population of 95,599 cattle, 141,882 poultry in the rural area and 24,015 cattle, 66,839 poultry in the urban area with the total of 119,614, 208,721 cattle and poultry population [1].

Zoonosis diseases which are naturally transmitted from vertebrate animals to human beings [2]. Approximately, 60% of all microbial agents of human beings are shared in nature with other animals [3]. Emerging and reemerging zoonotic diseases having a potentially dangerous impact on human health have brought worldwide attention to them [4]. Due to climatic changes, the incidence of emerging and reemerging diseases has increased to a greater extent [5].

The objective of veterinary public health is to improve human health using the knowledge of veterinary science. Salmonellosis, *Escherichia coli*, campylobacteriosis, and listeriosis which are associated with the current foodborne disease outbreak are of major concern in developing countries. In addition to it, zoonotic diseases such as brucellosis, leptospirosis, rabies, bovine TB, hydatidosis, cysticercosis, taeniasis, and toxoplasmosis need attention of veterinary public health service [6].

Animal disease such as anthrax, cysticercosis, brucellosis, bovine TB, rabies, and hydatidosis has an important zoonotic potential [7]. Diseases can also be transmitted to humans through contamination during production, processing, and handling of animal products. Other risk factors contributing to zoonotic outbreaks are working with diseased animals, skinning and slaughtering of infected animals, improper disposal of animal waste, and infective materials of diseases animals. Lack of awareness among livestock owners is the important cause of zoonotic diseases and it is also an important hurdle in controlling zoonotic diseases [8].

Zoonotic diseases have a great impact on livelihood of livestock farmers by affecting their health and reducing the quantity and quality of animal products thereby causing huge economic loss, further economy will be impaired and loss of livestock product market because of decreased consumer confidence [9-11]. Lack of awareness and knowledge about the zoonotic disease reported to be associated with the occurrence of zoonotic disease in humans [12,13]. An extensive program was implemented by WHO in controlling rabies in India. Due to this program, economic loss due to rabies in India has drastically come down. A similar type of program if implemented for diseases like leptospirosis and brucellosis, economic losses caused by those diseases in terms of human health and animal health can also be brought down. To implement such program understanding about public knowledge, awareness and animal husbandry practices could be a useful tool in implementing a disease awareness and control program [2,14,15]. Hence, this study was undertaken to study the extent of knowledge, awareness, attitude, and risks of zoonotic diseases among livestock owners in Puducherry region.

## Materials and Methods

### Ethical approval

Ethical approval was not required in this survey based study; however, the data were collected after obtaining consent from all the participants.

### Sampling area and size

**Figure 1:**
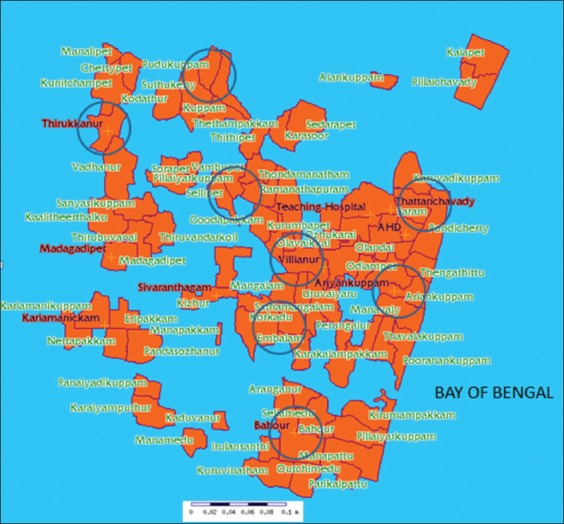
Geographical information system map of Puducherry showing 81 revenue villages and sampling area represented by a circle.

Puducherry union territory has a total of 81 revenue villages from which a total of 250 livestock farmers were selected randomly from eight revenue villages. And each farmer was interviewed with a questionnaire. Geographical information system map showing 81 revenue villages and area selected for the study was shown in Figure-1.

### Data collection

The questionnaire containing both open- and close-ended questions on various aspects of zoonotic diseases, i.e., awareness, knowledge, risks, animal waste disposal, and personal hygiene was used to interview the respondents. A total of 49 questionnaires were framed to assess the source and transmission of infection to the farmers and to test their knowledge and awareness about zoonotic diseases. The information about independent variables, *viz*., education, income, age, animal waste disposal, and herd size was collected with the help of structured schedule and scales.

### Statistical analysis

The data collected were analyzed by chi-square test using software Graph pad prism, and results were used to assess the relationship between education level and zoonotic disease awareness; risk of zoonotic diseases and its relation with independent variables.

## Results

### Education and socioeconomic status of the livestock farmers

**Figure 2:**
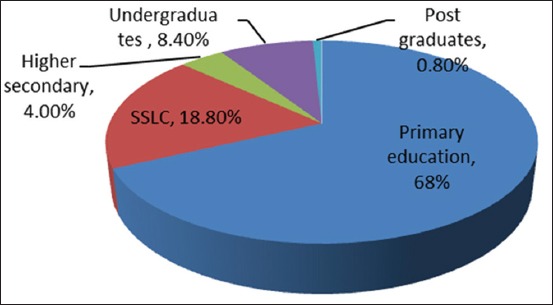
Educational qualification of the respondents (farmers).

Based on the study conducted in Puducherry, it has been found that the majority of farmers involved in livestock rearing are primarily educated (Figure-2). Moreover, the majority of them are able to read and write in their mother tongue.

The present survey analysis represents that most of the respondents are belonging to the age group of 41-60 years (Table-1) and many of them are women since they do not like to go out and work. And livestock rearing is considered as one of the most important sources of livelihood by the farmers.

**Table-1:** Respondents age.

Age (years)	Frequency (%)	95% confidence interval
<25	19 (7.6)	4.92-11.56
26-40	62 (24.8)	19.86-30.51
41-60	115 (46)	39.93-52.19
>60	54 (21.6)	16.95-27.11
Total	250 (100)	-

About 42.8% of respondents’ household having a graduate which in turn indicates that easy accessibility to higher level of education is present in rural people of Puducherry (Table-2).

**Table-2:** Highest education in the family members.

Category	Education	Frequency (%)	95% Confidence interval
1.	Primary education	50 (20)	15.51-25.4
2.	SSLC	47 (18.8)	14.44-24.10
3.	Higher secondary	46 (18.40)	14.09-23.67
4.	Undergraduates	86 (34.4)	28.79-40.48
5.	Post graduates	21 (8.4)	5.56-12.50
	Total	250 (100)	-

The present investigation reveals that most of the respondents are small-scale farmers and their monthly income was less than Rs. 10,000 which makes living more arduous (Table-3).

**Table-3:** Average monthly income of the respondents’ family.

Income/month	Frequency (%)	95% confidence interval
≤10,000	198 (79.2)	73.75-83.77
10,000-20,000	41 (16.4)	12.33-21.49
≥20,000	11 (4.4)	2.47-7.71
Total	250 (100)	-

### Awareness, knowledge and risk factors associated with animal management toward zoonotic diseases

**Figure 3:**
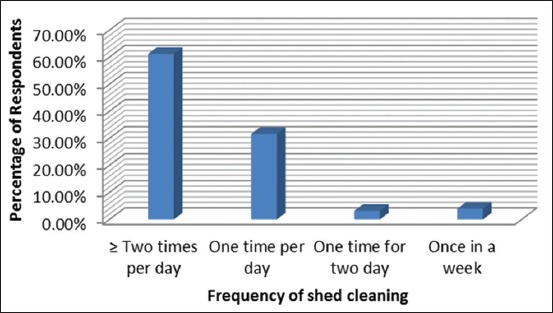
Frequency of shed cleaning.

About 61.2% of farmers were keeping their animal shed clean, implicating their traditional way of maintaining the animals. This also indicates less chance of getting many diseases from the animals by the livestock farmers (Figure-3).

Various awareness programs implemented by Puducherry government toward control of rabies was reflected on by the majority of the respondents (48%), who suggested that for dog bitten wound, immediate action was to be taken by cleaning the wound with soap. But still 29.6% of the respondents were ignorant about cleaning the dog bitten wound. Around 1.2% of the respondent still suggests application of chili powder on the dog bitten wound (Table-4).

**Table-4:** First aid proposed by respondents’ for a dog bite wound.

Method of first aid	Frequency (%)	95% confidence interval
Wash with water	48 (19.2)	14.80-24.53
Wash with soap	122 (48)	42.67-54.97
Put chilly powder	3 (1.2)	0.41-3.47
No washing	74 (29.6)	24.28-35.53
Total	250 (100)	-

As for the awareness toward zoonoses is concerned, only 16.4% of respondents knew that diseases in animals can be transmitted to humans. Of those respondents, 51.29% of them knew some specific zoonotic diseases. Majority of them knew that diarrheic diseases in livestock can be contracted by them. Three respondents mentioned foot-and-mouth disease (FMD) as a potential zoonotic disease.

About 4.8%, 3.6%, 6.8%, and 22.4% of respondents knew about the zoonotic potential of diseases like brucellosis, TB, anthrax, and avian flu, respectively. Among 28 respondents who own dogs, only 14.3% were doing proper deworming. Calf hood vaccination against brucellosis was known only to 0.8% of the respondents (Table-5).

**Table-5:** Awareness about zoonotic disease by respondents’ of Puducherry region.

Facts known/activity done	Frequency (%)
Disease can transmit from animals→Humans	41 (16.4)
Specific zoonoses known	21 (8.4)
Brucellosis known	12 (4.8)
Wash with soap on dog bitten wound	122 (48)
Cattle can get TB	24 (9.6)
TB from livestock→Humans	9 (3.6)
TB from humans→Livestock	15 (6)
Testing livestock for TB	11 (4.4)
Anthrax from livestock→Humans	17 (6.8)
Vaccination for brucellosis	2 (0.8)
Deworming of pet dog among dog owners	4 (14.3)
Avian flu from poultry→Humans	56 (22.4)

TB=Tuberculosis

In this study, about 43.2% of the responded reported FMD outbreak in their cattle and 24.07% reported hand and foot lesion after attending the FMD infected animals (Table-6).

**Table-6:** Zoonotic potential of FMD among livestock owners in Puducherry region.

Parameter	Frequency (%)
FMD occurrence	108 (43.2)
Hand lesions among owners handled FMD animal	26 (24.07)

FMD=Foot-and-mouth disease

Regarding the list of zoonotic diseases contracted by the livestock owners in Puducherry region, 18% of the respondents were aware about zoonotic diseases from cattle. Among the respondents who have contracted zoonotic diseases, 37.7% reported respiratory infection, 31.1% digestive disturbances, 15.5% had dermatological problem and 15.5% reported indiscrete disease such as fever, body pain, headache, and joint pain. From the respondents who have got the zoonotic disease (n=45), 51.2% of the respondent reported chronic infection and 48.8% of the respondent reported acute form of zoonotic infection (Table-7).

**Table-7:** List of zoonotic diseases contracted by the livestock owners in Puducherry region.

Parameter	Frequency	Percentage
Got disease from animal	45	18
Got respiratory disease	17	37.7 (n=45)
Got digestive disease	14	31.1 (n=45)
Got skin disease	7	15.5 (n=45)
Got other forms of disease	7	15.5 (n=45)
Disease for<1 week	16	35.5 (n=45)
Disease for 2-3 weeks	6	13.3 (n=45)
Disease for 1 month	2	4.5 (n=45)
Disease for>1 month	21	46.7 (n=45)

**Figure 4:**
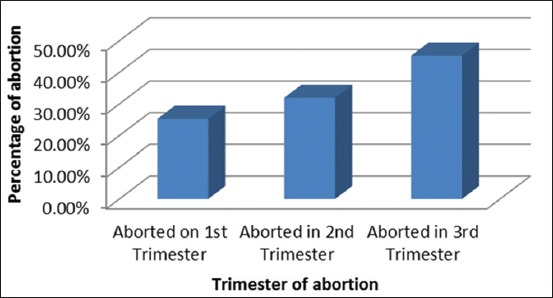
Occurrence of abortion in the Livestock’s and the trimester of abortion.

In this study, the facts were revealed that in about 30% of the respondents’ farm had an incidence of abortion. Among them, 25.3%, 32% and 45.3% of them noted abortions at 1^st^, 2^nd^ and 3^rd^ trimester of gestation, respectively (Figure-4).

### Relationships between different variables analyzed by Chi-square test

Different independent variables were analyzed by chi-square test and Fisher’s exact test and data and statistical significance were represented in Table-8. The data revealed that there was no significance in the income of the respondent and their method of disposal of carcasses; frequency of livestock shed cleaning and incidence of disease symptoms among respondents; habit of sleeping inside cattle shed and incidence of disease symptoms among respondents; education level of dog owners and deworming, vaccination of their pets.

**Table-8:** Relationships between different variables analyzed by Chi-square test.

S. No.	Variable 1	Variable 2			P value

	Income of respondents	Method of disposal of carcass			

		Proper disposal	Improper disposal	Total
1.	Income<10,000	39	12	51	0.1331
	Income>10,000	127	71	198	
	Total	166	83	249	

	**FMD incidence in respondents’ cattle**	**Occurrence of hand and foot lesions among respondents**			

		**FMD positive**	**FMD negative**	**Total**

2.	Hand lesions positive	17	9	26	0.0208*
	Hand lesions negative	91	133	224	
	Total	108	142	250	

	**Frequency of livestock shed cleaning**	**Incidence of disease symptoms among respondents**			
	
	**Frequency of shed cleaning**	**Zoonoses positive**	**Zoonoses negative**	**Total**

3.	>1 time per day	41	191	232	0.5412
	<3 times per week	4	14	18	
	Total	45	205	250	

	**Educational level of dog owners**	**Deworming their dogs**			
	
	**Education level**	**Dewormed**	**Not dewormed**	**Total**

4.	HSS graduates	2	2	4	0.0856
	Primary, SSLC	2	22	24	
	Total	4	24	28	

	**Habit of sleeping inside cattle shed**	**Incidence of disease symptoms among respondents**			

		**Zoonoses positive**	**Zoonoses negative**	**Total**

5.	Sleeping inside cattle shed	6	13	19	0.1221
	Not sleeping inside cattle shed	39	192	231	
	Total	45	205	250	

	**Educational level of dog owners**	**Vaccinating their dogs**		
	
	**Education level**	**Vaccinated**	**Not vaccinated**	**Total**

6.	HSS, graduate	3	1	4	0.285
	Primary, SSLC	9	15	24	
	Total	12	16	28	

	**Educational level of respondents**	**Treatment of dog bitten animals**		
	
	**Education level**	**Treatment given**	**Treatment not given**	**Total**

7.	Primary, SSLC	217	33	250	<0.0001**
	HSS, graduate	171	79	250	

** Significant (p≤0.01),

* Significant (p≤0.05), ^+^Significant (p≤0.10), ns=Non-significant, FMD=Foot-and-mouth disease

Further our analyses showed that there was a significant relationship in the educational level of respondents and treatment of dog bitten animals. Furthermore, there was statistical significance in occurrence of hand and foot lesions in the respondent and occurrence of FMD outbreak in their animals.

## Discussion

The interface among people, animal and the surrounding environment is very close in many developing and developed countries, where animals act as a companion and provide draught power, transportation, clothing, fuel and source of protein in the form of milk, meat and eggs. In the absence of proper care and lack of awareness, this linkage can lead to a serious risk to public health with huge economic penalties [16]. Studying the community socioeconomic status, education and perception of the community on various zoonotic diseases and its risk is a crucial step toward the development and implementation of suitable disease prevention and control strategies.

Currently, there is no documented evidence available on the awareness of zoonoses among the rural and urban communities in the Puducherry region. Hence, this study was undertaken to assess the awareness on zoonotic diseases among livestock farmers.

### Education and socioeconomic status of the livestock farmers

Based on the study conducted in Puducherry, it has been found that majority of farmers involved in livestock rearing are primarily educated (Figure-2). Moreover, the majority of them are able to read and write in their mother tongue.

As for the awareness toward zoonoses is concerned, only 16.4% of respondents knew that diseases in animals can be transmitted to humans. About 4.8%, 3.6%, 6.8%, and 22.4% of respondents knew about the zoonotic potential of diseases such as brucellosis, TB, anthrax, and avian flu, respectively. This study indicated a relatively lower level of awareness of the respondents in the study area. Similar lower levels of awareness were also reported by other workers [17,18]. This study is in conjunction with the work of others [19] who reported that a high number of farmers had no thorough and accurate knowledge about zoonotic diseases. Some research workers reported [20] higher level of zoonotic awareness of the respondents in Addis Ababa. The difference in the above two study could be due to dissimilarity in the provision of information about these disease and food habits, etc. [18]. Lack of knowledge on zoonotic diseases is due to poor communication between veterinarian and human health-care professionals [21]. This low level of knowledge and awareness on zoonoses is likely to expose livestock farmers to increased risk of zoonotic diseases.

In this study, regarding the list of zoonotic diseases contracted (n=45), 37.7% reported respiratory infection, 31.1% digestive disturbances, 15.5% had dermatological problem and 15.5% reported indiscrete disease such as fever, body pain, headache, and joint pain. The present reports clearly indicate a lack of awareness about the zoonotic diseases.

On the other hand in this study, awareness about rabies was high and 48% of the respondent had better knowledge about rabies and its management and our analysis showed significance in educational level of respondents and treatment of dog bitten animals. Our present findings are in agreement with another researcher who also has reported the similar findings [15,22].

Like rabies, brucellosis is a very important zoonotic disease and one of the important causative agents of abortion in livestock and is a highly infectious zoonotic disease. In Brucellosis, the classical signs are abortion in the 3^rd^ trimester of gestation and incidence of retained placenta [23]. In this study, about 30% of the respondents’ farm had an incidence of abortion. Among them, 25.3%, 32% and 45.3% of them noted abortions at 1^st^, 2^nd^ and 3^rd^ trimester of gestation, respectively. However, only 4.8% of the respondent knows about brucellosis and only 0.8 % of the respondent knows about vaccination against brucellosis. This may be due to lack of awareness against brucellosis. However, many respondents reported 3^rd^ trimester abortion in their cattle and hence awareness of brucellosis is a need of an hour for control of highly potential zoonotic diseases like brucellosis, knowledge about calf hood vaccination must be initiated to the livestock farmers.

In this study, about 43.2% of the respondents reported FMD outbreak in their cattle and 24.07% reported hand and foot lesion after attending the FMD infected animals which was statistically significant. Another worker [24] also reported the similar findings that the humans are believed to be slightly susceptible to infection with the FMD virus.

**Figure 5:**
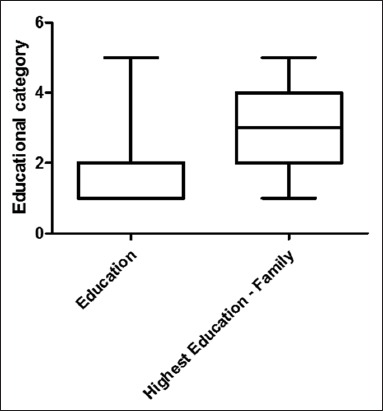
A box and whiskers plot illustrating Education of the respondents to the highest education in the respondents family. The bold line indicates the median. Whiskers represent the 5^th^ and 95^th^ percentiles with lines below and above representing the 0-5^th^ and 95-100^th^ percentiles, respectively. Outliers are indicated by individual points (Category: 1 - Primary education; 2 - SSLC; 3 - Higher secondary; 4 - Undergraduates; 5 - Post-graduates).

In this study, level of education in the family member was higher when compared to the level of education in the persons who is carrying out farming practices (Figure-5). Hence if the educated family member also involves themselves in the farming practices, implementation of zoonotic awareness and control programs will be effective and easier.

## Conclusion

Lack of awareness about the zoonotic diseases in the present investigation was due to poor communication between veterinarian and human health-care professionals and lack of involvement of educated family members in farming activities. Involvement of educated family members in farming practices can solve this issue. Further creation of zoonotic disease awareness among livestock farmers is of utmost important. Proper disposal of animal waste, good hygienic practices, are extremely important steps in successful control of zoonotic diseases [25].

## Authors’ Contributions

KR: Revised the questionnaire for collection of data, framed manuscripts and carried out statistical analysis; AB: Provided valuable suggestions regarding the study design and analysis of the collected data; SD, SHB, RH, MJ and NB: Prepared questionnaire, assisted in framing manuscripts, collected data by personal interview, recorded the data and prepared tables. All authors read and approved the final manuscript.
